# Development and evaluation of a risk prediction model for diabetes mellitus type 2 patients with vision-threatening diabetic retinopathy

**DOI:** 10.3389/fendo.2023.1244601

**Published:** 2023-08-24

**Authors:** Di Gong, Lyujie Fang, Yixian Cai, Ieng Chong, Junhong Guo, Zhichao Yan, Xiaoli Shen, Weihua Yang, Jiantao Wang

**Affiliations:** ^1^ Shenzhen Eye Hospital, Jinan University, Shenzhen, Guangdong, China; ^2^ The First Affiliated Hospital of Jinan University, Jinan University, Guangzhou, Guangdong, China; ^3^ Macau University Hospital, Macao, Macao SAR, China; ^4^ Shenzhen Eye Hospital, Jinan University, Shenzhen Eye Institute, Shenzhen, Guangdong, China

**Keywords:** diabetes mellitus type 2, diabetic retinopathy, vision-threatening diabetic retinopathy, risk factors, prediction model

## Abstract

**Objective:**

This study aims to develop and evaluate a non-imaging clinical data-based nomogram for predicting the risk of vision-threatening diabetic retinopathy (VTDR) in diabetes mellitus type 2 (T2DM) patients.

**Methods:**

Based on the baseline data of the Guangdong Shaoguan Diabetes Cohort Study conducted by the Zhongshan Ophthalmic Center (ZOC) in 2019, 2294 complete data of T2DM patients were randomly divided into a training set (n=1605) and a testing set (n=689). Independent risk factors were selected through univariate and multivariate logistic regression analysis on the training dataset, and a nomogram was constructed for predicting the risk of VTDR in T2DM patients. The model was evaluated using receiver operating characteristic (ROC) curves and area under the curve (AUC) in the training and testing datasets to assess discrimination, and Hosmer-Lemeshow test and calibration curves to assess calibration.

**Results:**

The results of the multivariate logistic regression analysis showed that Age (OR = 0.954, 95% CI: 0.940-0.969, *p* = 0.000), BMI (OR = 0.942, 95% CI: 0.902-0.984, *p* = 0.007), systolic blood pressure (SBP) (OR =1.014, 95% CI: 1.007-1.022, *p* = 0.000), diabetes duration (10-15y: OR =3.126, 95% CI: 2.087-4.682, *p* = 0.000; >15y: OR =3.750, 95% CI: 2.362-5.954, *p* = 0.000), and glycated hemoglobin (HbA1C) (OR = 1.325, 95% CI: 1.221-1.438, *p* = 0.000) were independent risk factors for T2DM patients with VTDR. A nomogram was constructed using these variables. The model discrimination results showed an AUC of 0.7193 for the training set and 0.6897 for the testing set. The Hosmer-Lemeshow test results showed a high consistency between the predicted and observed probabilities for both the training set (Chi-square=2.2029, *P*=0.9742) and the testing set (Chi-square=7.6628, *P*=0.4671).

**Conclusion:**

The introduction of Age, BMI, SBP, Duration, and HbA1C as variables helps to stratify the risk of T2DM patients with VTDR.

## 1 Background

According to research conducted by the International Diabetes Federation (IDF), the global prevalence of diabetes was estimated to be 9.3% (463 million people) in 2019, with a predicted increase to 10.2% (578 million people) by 2030 and 10.9% (700 million people) by 2045 (1). Diabetic retinopathy (DR), a highly tissue-specific neurovascular complication of diabetes, is the leading cause of preventable blindness in the working-age population (2). In addition to vision loss, DR has been shown to be associated with other diabetes-related complications, including kidney disease, peripheral neuropathy, and cardiovascular events (3–5). Therefore, targeted monitoring and management of DR patients are of crucial clinical significance.

The International Clinical Diabetic Retinopathy Disease Severity Scale categorizes DR into five stages based on disease severity, including the first three stages with low risk, as well as the fourth stage, severe non-proliferative diabetic retinopathy (NPDR), and the fifth stage, proliferative diabetic retinopathy (PDR). Diabetic macular edema (DME) is classified as either present or absent (6). Vision-threatening diabetic retinopathy (VTDR) includes severe NPDR, PDR, and/or DME, indicating that the development of DR has seriously affected the patient’s vision, and failure to treat it in a timely manner will result in irreversible vision loss (7). The pathogenesis of DR is still not fully understood, but it may be due to excessive production of reactive oxygen species (ROS) and advanced glycation end-products (AGEs) inducing mitochondrial dysfunction, leading to dysfunction of the vascular endothelial cell barrier, neuronal cell death, and axonal degeneration, ultimately resulting in severe damage to retinal function (8). Studies have found that risk factors for DR and VTDR may include race, place of residence, refractive error, duration of diabetes, blood glucose levels, blood pressure, and kidney function, and optimizing control of these risk factors can reduce the risk of VTDR occurrence and progression (9–12).

The main treatments for VTDR include panretinal photocoagulation, intravitreal injection of anti-vascular endothelial growth factor, and vitreoretinal surgery (2). Primary healthcare providers, optometrists, and nurses who have received training can effectively manage mild to moderate DR, but management and key treatment of VTDR patients require the specialized knowledge and skills of trained ophthalmologists or retinal specialists (13, 14). According to statistics, the prevalence of VTDR in the global diabetic population is 6.17% (285.4 million people), and it is expected to increase to 448.2 million people by 2045 (7). In recent years, artificial intelligence diagnostic systems have demonstrated excellent performance in the identification and referral of VTDR patients (15, 16). However, most autonomous AI systems currently require imaging data provided by advanced ophthalmic examination equipment, such as fundus photography and OCT, and advanced ophthalmic medical resources are still scarce for physicians and patients in underdeveloped areas (14).

Logistic regression, as a commonly used statistical method, can be applied in various clinical medical scenarios, including but not limited to the following aspects: 1)Disease Prediction: Logistic regression can predict whether patients have a certain disease based on their clinical data. 2)Risk Assessment: Logistic regression can be utilized to assess the risk of patients developing a specific disease. 3)Diagnostic Assistance: Logistic regression can assist physicians in disease diagnosis. 4)Drug Development: In the drug development process, logistic regression can predict the efficacy and safety of new drugs. By establishing models, researchers can evaluate the therapeutic effects of different drugs on diseases and identify the most promising candidates. 4)Survival Analysis: In clinical observations and epidemiological studies, logistic regression can be used to analyze patients’ survival data, predict their survival rates, and identify factors that affect survival. Overall, logistic regression plays a crucial role in clinical medicine, providing an effective predictive and decision-making tool for physicians and researchers to improve patient health outcomes and the quality of medical services.

In clinical prediction models, logistic regression analysis is commonly used for predicting the onset and diagnosis of diseases by analyzing the probability of an individual developing a certain outcome event at different values of the predictive indicator. Through logistic regression analysis of multiple factors, independent influencing factors for the outcome event are identified as predictive indicators in the prediction model. Then, multiple predictive indicators are integrated and analyzed using regression analysis to generate a risk nomogram based on a certain proportion (17).

Currently, there are few clinical prediction model studies on DR and VTDR based on non-imaging data. Ke et al. constructed a risk nomogram for predicting the development of VTDR in mild NPDR patients by introducing three variables, including 2-hour C-peptide, UACR, and sural nerve conduction impairment (SNCI). The model achieved a sensitivity, specificity, and AUC of 66.7%, 89.5%, and 0.75, respectively, in the testing set (18).

In most areas of China, there are standard management and regular physical examination protocols for diabetic patients. However, the screening and management system for diabetic retinopathy (DR) remains incomplete. Therefore, the purpose of this study is to develop and validate a VTDR risk prediction scoring model based on systemic parameters and non-ophthalmic imaging data, using the T2DM cohort study data. The goal is to provide a simple scoring system for regions with limited medical resources and primary healthcare institutions. This system can be used to identify high-risk individuals for VTDR, addressing the gaps in existing medical facilities and enhancing healthcare accessibility for at-risk populations.

## 2 Materials and methods

### 2.1 The main workflow of this study

**Figure 1:**
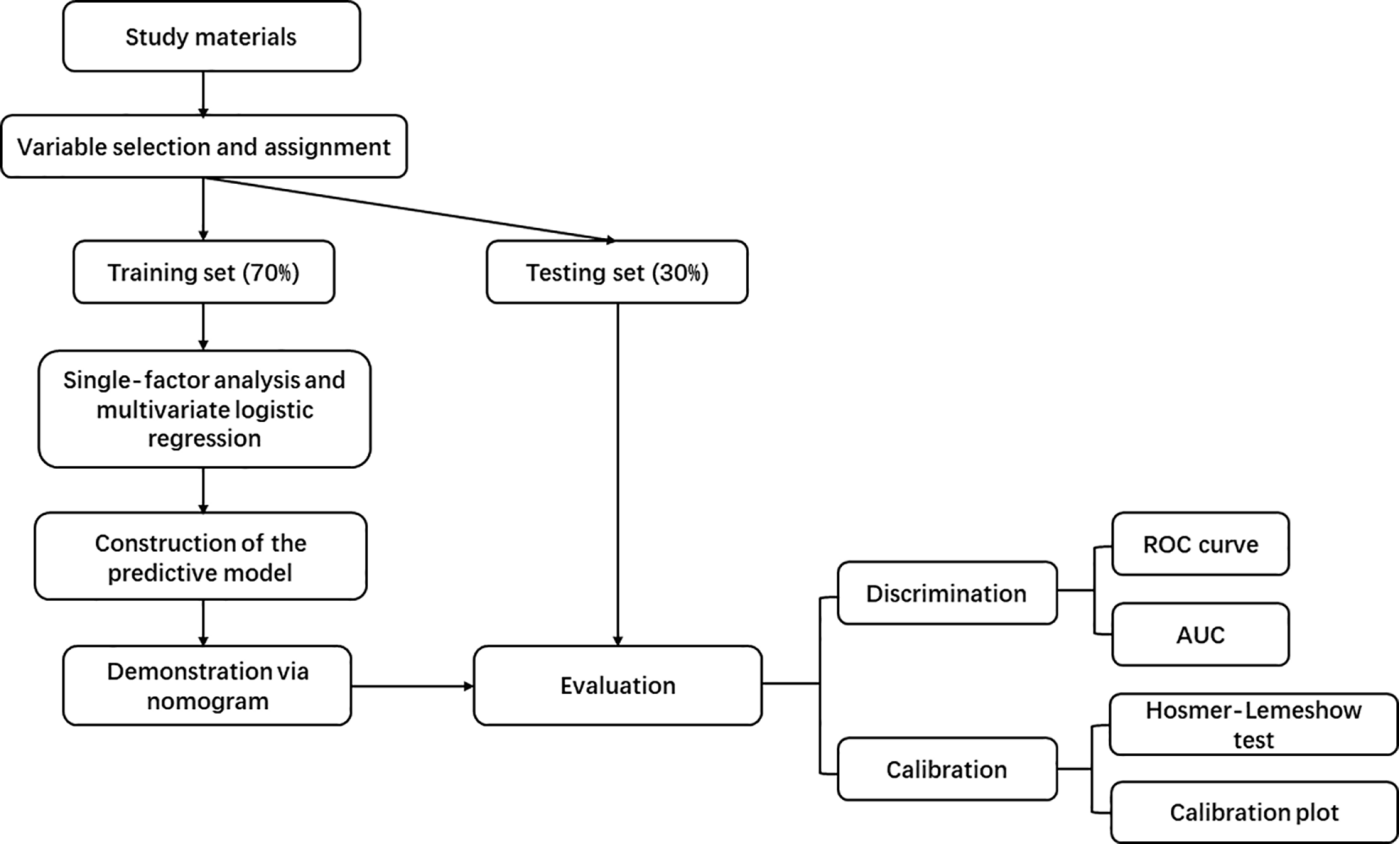
Workflow Diagram.

The main workflow of this study summarized in **Figure 1**.

### 2.2 Study materials

a) The baseline data of 2677 patients were collected from the Guangdong Shaoguan Diabetes Cohort Study conducted by Zhongshan Ophthalmic Center (ZOC), Sun Yat-sen University in Guangzhou, China in 2019.

b) All patients were diagnosed with T2DM.

c) All patients underwent fundus photography and OCT examinations, and experienced ophthalmologists diagnosed and verified DR and DME. Patients with severe NPDR, PDR, and/or DME were defined as having VTDR.

### 2.3 Variable selection

PubMed, EMBASE, and other websites were searched using keywords such as “T2DM” and “VTDR” to identify relevant literature and determine the variables to be included in the study. The following patient data were extracted: ID number, Age, Gender, body mass index (BMI), systolic blood pressure (SBP), diastolic blood pressure (DBP), duration of diabetes(Duration), smoking status(Smoking), alcohol consumption(Alcohol), alanine aminotransferase (ALT), aspartate aminotransferase (AST), blood urea nitrogen (BUN), serum creatinine (SCr), uric acid (UA), total cholesterol (TC), triglycerides (TG), high-density lipoprotein (HDL), low-density lipoprotein cholesterol (LDL-C), blood glucose (GLU), glycated hemoglobin (HbA1C), and whether VTDR occurred. The extracted data were organized and merged into one file based on the patient ID number.

### 2.4 Variable assignment explanation

All categorical variables, including gender, duration of diabetes, smoking, alcohol consumption, residence, and whether VTDR occurred, were assigned values. The assigned values are shown in **Table 1**.

**Table 1:** Variable Assignment Explanation.

Variable name	Variable Meaning	Assignment Explanation
Gender	Gender	1=Male
		2=Female
Duration	Duration of diabetes	1=<5y
		2 = 5~10y
		3 = 10~15y
		4= >15y
Smoking	Smoking	0=No
		1=Yes
Alcohol	Alcohol consumption	0=No
		1=Yes
Resident	Location of residence	1=Rural
		2=Urban
		3=Both urban and rural
VTDR	Vision-Threatening Diabetic Retinopathy	0=No/Unknow
		1=Yes

### 2.5 Construction and presentation of the predictive model

Logistic regression analysis is a statistical technique used to analyze the relationships between multiple variables, where the outcome variable is a categorical variable, including binary, unordered categorical, and ordered categorical variables. The number and types of independent variables are not limited. In this study, the well-organized data was randomly divided into training and testing sets in a 7:3 ratio. Single-factor analysis was conducted on all variables in the training set, and variables with a p-value< 0.05 were included in the multivariable logistic regression to identify independent influencing factors. The multivariable logistic regression is based on the logistic regression model, which establishes a linear equation to describe the relationship between independent variables and log odds. The log odds are then converted to probabilities for predicting and interpreting the outcome categories. The logistic regression model can be represented by the following equation:


$$\text{logit}\left(\text{p}\right)=\beta 0+\beta 1X1+\beta 2X2+\ldots +\beta n*Xn_{˚}$$

logit(p) represents the log odds, p is the probability of the event occurring, X1, X2,…, Xn are the independent variables, and β0, β1, β2,…, βn are the model parameters (coefficients). By estimating the parameter values, the best-fitting model can be obtained to predict the probability of the outcome variable.

We establish a predictive model to identify independent influencing factors of VTDR in patients with T2DM and present it in the form of a Nomogram.

### 2.6 Evaluation of the predictive model

The ROC curve and AUC was used to evaluate the discrimination of the predictive model, with an AUC range of 0-1, where 1 indicates complete consistency and 0.5 indicates poor consistency. The Hosmer-Lemeshow test and calibration plot were used to calibrate the predictive model and judge the consistency between the predicted probability and the observed probability. When P>0.05 for the Hosmer-Lemeshow test, it can be considered that the predictive model has good calibration.

The calibration plot is a commonly used visual tool for assessing the consistency between model-predicted probabilities and actual observed outcomes. In the calibration plot, the x-axis represents the predicted probabilities from the model, while the y-axis represents the frequency or probabilities of the observed outcomes. The predicted results refer to the model’s outputs obtained by predicting new, unseen samples based on input features and learned parameters. On the other hand, the observed outcomes represent the known class labels of the actual observed samples, which are used to compare the accuracy and effectiveness of the model’s predictions.

## 3 Statistical methods

Continuous variables that conform to normal distribution are expressed as mean and SD values, while other continuous variables are expressed as median (25-75 percentile). Categorical variables are expressed as percentages. Single-factor analysis and multivariate logistic regression were used to eliminate variables with limited predictive ability. Logistic regression was used to establish a predictive model, and a Nomogram was used to predict the incidence rate. All data were analyzed using SPSS version 27.0 and R version 4.2.2 software, and *P*<0.05 was considered statistically significant.

## 4 Results

### 4.1 Detection of VTDR in patients with T2DM

A total of 2677 patients with T2DM were collected and summarized. Individuals with missing variables were excluded, resulting in a final sample of 2294 patients with T2DM. Among them, 370 patients were diagnosed with VTDR, with a prevalence of 16.13%.

### 4.2 The statistical description of the training set and testing set

The original dataset consists of 2294 cases, which were randomly divided into a training set and a testing set. The training set includes 1605 cases (70%), and the testing set includes 689 cases (30%). The distribution differences of the 21 variables between the two groups were not statistically significant (P>0.05), as shown in **Table 2**.

**Table 2:** Statistical description of the training and testing sets.

Variable	Classification	Data set		*t*-value/*Z*-value/*X^2^*-value	*P*-value
		Training set (n=1605)	Testing set (n=689)		
Age		63.37 ± 10.13	63.58 ± 9.69	-0.463	0.643
Gender	1(Male)	718(70.9%)	294(29.1%)	0.834	0.361
	2(Female)	887(69.2%)	395(30.8%)		
BMI		24.37 ± 3.43	24.21 ± 3.30	0.823	0.411
SBP		136.92 ± 18.52	137.56 ± 18.92	-0.755	0.450
DBP		81.94 ± 11.06	82.53 ± 11.09	-1.168	0.243
Duration	1(<5y)	837(69.6%)	365((30.4%)	5.060	0.167
	2(5~10y)	424(67.7%)	202(32.3%)		
	3(10~15y)	200(73.0%)	74(27.0%)		
	4(>15y)	144(75.0%)	48(25.0%)		
Smoking	0(No)	1317(70.0%)	565(30.0%)	0.001	0.976
	1(Yes)	288(69.9%)	124(30.1%)		
Alcohol	0(No)	1392(69.4%)	613(30.6%)	2.198	0.138
	1(Yes)	213(73.7%)	76(26.3%)		
Resident	1(Rural)	918(70.1%)	392(29.9%)	0.063	0.969
	2(Urban)	679(69.8%)	294(30.2%)		
	3(Both urban and rural)	8(72.7%)	3(27.3%)		
ALT		19.00(14.60, 26.05)	18.60(14.10, 25.50)	-1.465	0.143
AST		23.00(19.20, 27.40)	22.20(19.00, 27.40)	-1.370	0.171
BUN		5.72(4.70, 6.93)	5.72(4.76, 7.02)	-0.961	0.337
SCr		90.70(78.80, 105.10)	88.30(78.40, 102.60)	-1.353	0.176
UA		353.78 ± 108.66	348.21 ± 107.38	1.129	0.259
TC		5.27 ± 1.10	5.36 ± 1.14	-1.810	0.070
TG		1.89(1.24, 3.06)	1.88(1.17, 2.89)	-0.978	0.328
HDL		1.20 ± 0.30	1.22 ± 0.29	-1.491	0.136
LDLC		2.92 ± 0.77	2.97 ± 0.78	-1.257	0.209
GLU		11.38 ± 5.49	11.42 ± 5.93	-0.148	0.882
HbA1C		7.46 ± 1.56	7.51 ± 1.60	-0.662	0.508
VTDR	0(No)	1342(69.8%)	582(30.2%)	0.261	0.609
	1(Yes)	263(71.1%)	107(28.9%)		

(t-test or non-parametric test for numerical variables; chi-square test for categorical variables;The t-value/Z-value/X^2^-value represent the statistics of t-test,non-parametric test and chi-square test respectively.)

### 4.3 Model variable selection:

#### 4.3.1 Univariate analysis

SPSS was used to perform statistical description on the relevant variables in the training set (1605 cases). The results showed that there were statistically significant differences (P<0.05) in 11 variables, including Age, BMI, SBP, DBP, Duration, ALT, AST, BUN, SCr, GLU, and HbA1C, between the two groups, as shown in **Table 3**.

**Table 3:** Results of Univariate analysis of the training set.

Variable	Classification	Whether concurrent VTDR		*t*-value/*Z*-value/*X^2^*-value	*P*-value
		Non-occurrence group (n=1342)	Occurrence group (n=263)		
Age		63.74 ± 10.19	61.48 ± 9.65	3.325	0.001***
Gender	1(Male)	597(83.1%)	121(16.9%)	0.206	0.650
	2(Female)	745(84.0%)	142(16.0%)		
BMI		24.43 ± 3.40	23.87 ± 3.55	2.420	0.016*
SBP		136.40 ± 18.31	139.57 ± 19.38	-2.542	0.011*
DBP		81.63 ± 10.99	83.50 ± 11.28	-2.512	0.012*
Duration	1(<5y)	743(88.8%)	94((11.2%)	58.584	0.000***
	2(5~10y)	356(84.0%)	68(16.0%)		
	3(10~15y)	142(71.0%)	58(29.0%)		
	4(>15y)	101(70.1%)	43(29.9%)		
Smoking	0(No)	1099(83.4%)	218(16.6%)	0.148	0.700
	1(Yes)	243(84.4%)	45(15.6%)		
Alcohol	0(No)	1166(83.8%)	226(16.2%)	0.174	0.677
	1(Yes)	176(82.6%)	37(17.4%)		
Resident	1(Rural)	766(83.4%)	152(16.6%)	0.125	0.939
	2(Urban)	569(83.8%)	110(16.2%)		
	3(Both urban and rural)	7(87.5%)	1(12.5%)		
ALT		19.30(14.90, 26.23)	18.50(13.40, 25.30)	-2.068	0.039*
AST		23.00(19.40, 27.50)	21.60(18.20, 27.00)	-2.769	0.006**
BUN		5.70(4.66, 6.83)	5.90(4.88, 7.34)	-2.794	0.005**
SCr		90.30(78.58, 103.20)	93.10(79.90, 113.10)	-3.049	0.002**
UA		355.06 ± 109.01	347.24 ± 106.81	1.068	0.286
TC		5.28 ± 1.11	5.24 ± 1.08	0.479	0.632
TG		1.90(1.25, 3.14)	1.83(1.18, 2.73)	-1.544	0.123
HDL		1.20 ± 0.30	1.20 ± 0.29	-0.074	0.941
LDLC		2.93 ± 0.78	2.89 ± 0.73	0.731	0.465
GLU		11.00 ± 5.33	13.33 ± 5.87	-6.380	0.000***
HbA1C		7.32 ± 1.49	8.19 ± 1.70	-8.482	0.000***

(t-test or non-parametric test for numerical variables; chi-square test for categorical variables;The t-value/Z-value/X^2^-value represent the statistics of t-test,non-parametric test and chi-square test respectively.) *p<0.01,**p<0.01,***p<0.001.

#### 4.3.2 Multivariable logistic regression analysis

The 11 variables identified through univariate analysis, including Age, BMI, SBP, DBP, Duration, ALT, AST, BUN, SCr, GLU, and HbA1C, were included in the binary logistic regression analysis using SPSS. The results showed that Age, BMI, SBP, Duration, and HbA1C (5 variables) were independent risk factors for VTDR in patients with T2DM (P<0.05), as shown in **Table 4**.

**Table 4:** Results of multivariate logistic regression analysis.

Variable		*B*-value	*P*-value	OR(95%CI)
Age		-0.047	0.000***	0.954(0.940-0.969)
BMI		-0.060	0.007**	0.942(0.902-0.984)
SBP		0.014	0.000***	1.014(1.007-1.022)
Duration	1(<5y)	–	0.000***	–
	2(5~10y)	0.336	0.065	1.399(0.980-1.997)
	3(10~15y)	1.140	0.000***	3.126(2.087-4.682)
	4(>15y)	1.322	0.000***	3.750(2.362-5.954)
HbA1C		0.282	0.000***	1.325(1.221-1.438)

(** p<0.01, *** p<0.001).

### 4.4 Construction of a predictive model for nomogram

**Figure 2:**
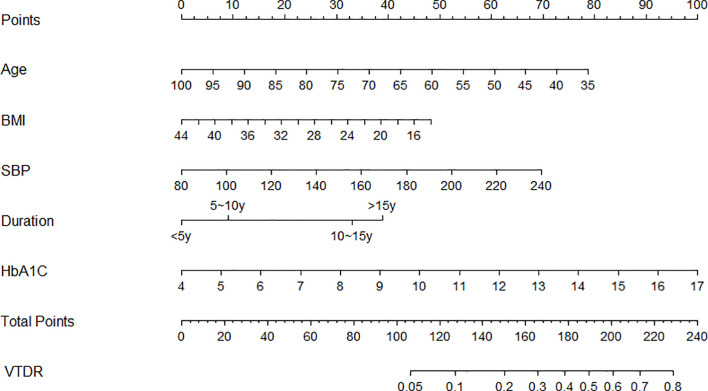
Nomogram prediction model for VTDR complications in T2DM patients.

This study used the multi-factor binary logistic regression method to select the predictive factors and built a nomogram model to predict the incidence of VTDR in T2DM patients using the “rms” package in Rstudio, based on the predictive factors selected from the multiple factor analysis (**Figure 2**). Each predictive variable corresponds to a set of numerical values, and the score is obtained by aligning the numerical values on the scale with the point scale at the top. The sum of all the scores corresponds to the total score, which is then aligned with the total point scale at the bottom to obtain the probability of VTDR in T2DM patients.

An example application is as follows: assuming that a 55-year-old (55 points) patient who has been diagnosed with type 2 diabetes mellitus for 10 years (32 points) has a BMI of 20 (40 points), a systolic blood pressure of 160 mmHg (34 points), and an HbA1C of 10% (45 points), his total score would be approximately 206, and the corresponding score would predict that the patient would have a probability of developing VTDR of about 65%.

### 4.5 Evaluation of the nomogram prediction model

#### 4.5.1 Discrimination

**Figure 3:**
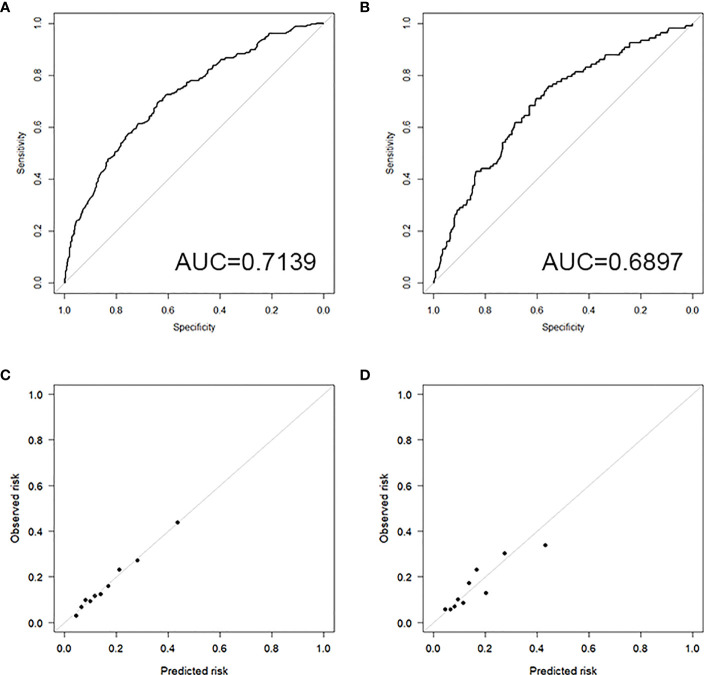
**(A)** ROC curve of the training set; **(B)** ROC curve of the testing set; **(C)** Calibration plot of the training set; **(D)** Calibration plot of the testing set.

The discrimination results show that the training set AUC is 0.7139 (**Figure 3A**), and the testing set AUC is 0.6897 (**Figure 3B**), indicating that the model has the ability to distinguish between the occurrence and non-occurrence of VTDR, which is helpful for risk stratification.

#### 4.5.2 Calibration

##### 4.5.2.1 Hosmer-Lemeshow test

The results of the Hosmer-Lemeshow test show a high level of consistency between the predicted probabilities and the observed probabilities in both the training set (Chi-square=2.2029, *P*=0.9742) and the testing set (Chi-square=7.6628, *P*=0.4671).

##### 4.5.2.2 Calibration plot

The calibration plots for both the training set (**Figure 3C**) and the testing set (**Figure 3D**) demonstrate good consistency between the predicted and observed outcomes, indicating that the model has good calibration.

## 5 Discuss

The statistical results of this study showed that Age, BMI, SBP, Duration, and HbA1C were independent influencing factors for VTDR in T2DM patients. Among them, the increase of SBP, Duration, and HbA1C was positively correlated with the onset of VTDR. It is worth noting that the increase of Age and BMI was negatively correlated with the onset of VTDR.

Regression analysis in this study showed an OR for Age of 0.954 (95% CI: 0.940-0.969, *p* = 0.000), i.e., for every 1-year increase in the age of an individual with T2DM, there was a 4.6% decrease in the risk of concurrent VTDR. This finding indicates that younger patients are at a higher risk of developing the disease compared to older patients. Contrasting with some earlier statistical studies which did not suggest age as a risk factor for DR, our results align with recent epidemiological studies (9, 10). Age, as well as the age of diabetes diagnosis, has been independently associated with macrovascular complications, including mortality, rather than microvascular complications (19, 20). For instance, Peng and Yu’s epidemiological study in Shenzhen, demonstrated a significant increase in the incidence of DR in patients over 60 years of age (21). Additionally, a cohort study on type 1 diabetes patients confirmed a correlation between the age of onset of type 1 diabetes and the occurrence and progression of DR, suggesting that older age at type 1 diabetes onset is associated with faster DR development (22). However, emerging evidence has shown that the diagnosis of type 2 diabetes in young people is linked to worse vascular risk features, higher incidence of complications, and a poorer prognosis (23, 24). Our statistical analysis supports this observation, indicating a higher risk of developing VTDR in younger patients, possibly due to poorer blood sugar control and more severe microvascular damage in this age group. Notably, evidence reveals that young patients exhibit worse blood sugar control compared to elderly patients, potentially owing to distinct phenotypes. Young patients mainly suffer from beta cell loss, whereas elderly patients experience a combination of insulin resistance and beta cell loss (20). In light of these findings, it is essential to conduct further research to clarify the complex relationship between age and DR/VTDR. Understanding the age-related factors influencing the risk of DR can contribute to improved preventive strategies and targeted treatment options.

The results of this study showed an OR for BMI of 0.942 (95% CI: 0.902-0.984, *p* = 0.007), i.e., for every 1-unit increase in BMI in individuals with T2DM, there was a 5.8% decrease in the risk of concurrent VTDR. Previous studies have found a possible neutral association between BMI and DR, indicating the existence of both protective and adverse effects (25). Han and X’s study showed that a higher BMI increased the risk of developing diabetes but was not related to VTDR (26). However, a large-scale epidemiological study conducted in India in 2022 demonstrated no relationship between BMI and DR, but it did reveal a negative correlation between BMI and VTDR (11). Remarkably, our study’s findings are consistent with the latter study, indicating that an increase in BMI is a protective factor for T2DM patients developing VTDR. Several reasons might account for this. Firstly, previous research has shown that individuals with T2DM and higher BMI tend to have elevated levels of C-peptide, which has been associated with a lower risk of DR (27, 28). Additionally, a higher BMI may reflect better blood sugar control and more aggressive treatment, contributing to the deceleration of DR development (25).

The logistic regression results showed an OR for SBP of 1.014 (95% CI: 1.007-1.022, *p* = 0.000), suggesting that for every 1-unit increase in SBP in individuals with T2DM, there was a 1.4% increase in the risk of concurrent VTDR. We found a positive correlation between the increase in SBP and the occurrence of VTDR, which is consistent with previous research (10, 29, 30). Specifically, studies have indicated that in Asian patients with well-controlled blood sugar, SBP variability is strongly linked to moderate DR (31). Additionally, a T2DM adult DR screening study in Chinese communities revealed that individuals with lower SBP (<140mmHg) had a significantly reduced risk of developing DR (32). Moreover, a large prospective cohort study of T2DM patients found that both systolic and diastolic blood pressure were associated with an increased risk of transitioning from the asymptomatic phase to mild DR and from mild DR to observable DR, suggesting that blood pressure may play a role in the early development of DR (33). The exact mechanism by which high blood pressure causes DR damage remains unclear. However, it is believed that sustained hypertension may lead to microvascular system structural damage, affecting retinal blood vessel endothelial cells, blood vessels, and surrounding tissues, resulting in retinal perfusion disorders. As a result, eyes with DR become more susceptible to excessive perfusion injury caused by hypertension, thereby accelerating the development of DR (34). Notably, current evidence supports that good blood pressure control is a controllable factor that not only reduces the probability of DR but also slows the comprehensive progression of already occurring DR (2, 33, 35). Additionally, studies have indicated that antihypertensive drugs may have a protective association with VTDR, possibly due to their protective effect on diabetes complications, in addition to their blood pressure-lowering effects (36).

As an indicator of cumulative microvascular system damage, the duration of diabetes has been shown to be positively correlated with the occurrence and development of DR and VTDR (10, 29, 30, 37). In patients with type 2 diabetes, it has been demonstrated that the duration of diabetes is independently associated with both macrovascular events and deaths and microvascular events. For every additional 5 years of diabetes, the adjusted risk of multiple microvascular events increases by 28% (20). However, as an uncontrollable factor, the duration of diabetes can only serve as a predictor and cannot be clinically intervened. Therefore, in high-risk populations, we can only reduce the risk of VTDR by controlling other controllable risk factors.

The study found an OR for HbA1C of 1.325 (95% CI: 1.221-1.438, *p* = 0.000), i.e., for every 1 percentage point increase in HbA1C in individuals with T2DM, there was a 32.5% increase in the risk of concurrent VTDR. Research has found that the optimal cutoff point for distinguishing HbA1C variables between patients with and without DR is 8.15% (38). However, there is currently no evidence to suggest clinical reference values for the critical point of HbA1C in relation to VTDR. Therefore, this study still considers HbA1C as a continuous variable for research. HbA1C reflects a patient’s blood glucose levels over the past 2-3 months, and its impact on the occurrence and progression of DR and VTDR has been thoroughly studied in previous research (9, 10, 29, 36, 37). The role of hyperglycemia in the occurrence and development of DR may be related to “metabolic memory,” which refers to the lasting adverse effects that high blood glucose has on the occurrence and progression of systemic complications. This mechanism may be due to the rapid changes in blood glucose control, which may not allow the retina enough time to recover from the damaging effects of previously high HbA1C levels (39). Studies have shown that when HbA1C is reduced from 8% to 7%, the risk of retinopathy will decrease by 30%-40% (40). Therefore, lowering HbA1C levels through medication or insulin therapy may be a reliable method for prevention and treatment.

After integrating all independent factors, this study included five variables, Age, BMI, SBP, Duration, and HbA1C, in the construction of a column chart model. Discrimination analysis showed that the training set AUC was 0.7193 and the testing set AUC was 0.6897, which was lower than Ke, J.’s prediction model (18). However, it should be noted that the variables used in Ke, J.’s model, including 2-hour C-peptide, UASCR, and SNCI, are not commonly used clinical observation indicators, indicating that their model may not be widely applicable in the real world (18). In contrast, this study included five clinical data that are relatively easy to obtain (Age, BMI, SBP, Duration, and HbA1C) as predictive factors in the model. Additionally, the Hosmer-Lemeshow test and calibration plot results of the model showed high consistency between the predicted and observed probabilities. Therefore, the comprehensive evaluation of this model is considered relatively ideal.

However, this study still has limitations. Although this study developed a new nomogram for T2DM complicated with VTDR, the modeling and validation data were both from epidemiological data in the same region and did not conduct application experiments in the real world. Therefore, this model still needs further improvement and validation.

## 6 Conclusion

Age, BMI, SBP, Duration, and HbA1C are independent factors that influence the development of VTDR in T2DM patients. Among them, the increase in SBP, Duration, and HbA1C is positively correlated with the incidence of VTDR, while the increase in age and BMI is negatively correlated with the incidence of VTDR. By introducing these 5 variables, the nomogram can help stratify the risk of VTDR in T2DM patients. However, further validation and improvement are still needed to enhance the reliability and generalizability of this model in real-world settings.

## Data availability statement

The original contributions presented in the study are included in the article/supplementary material, further inquiries can be directed to the corresponding author/s.

## Ethics statement

The studies involving humans were approved by Zhongshan Ophthalmic Centre Ethics Review Committee. The studies were conducted in accordance with the local legislation and institutional requirements. Written informed consent for participation in this study was provided by the participants’ legal guardians/next of kin.

## Author contributions

DG, LF, and YC: methodology, formal analysis, and writing – original draft. IC, JG, ZY, and XS: methodology and writing – review & editing. WY and JW: conceptualization, resources, writing – review & editing, and funding acquisition. All authors contributed to the article and approved the submitted version.
